# Assistive Technologies, Robotics, Automatic Machines: Perspectives of Integration in the *Health Domain*

**DOI:** 10.3390/healthcare10061080

**Published:** 2022-06-10

**Authors:** Daniele Giansanti

**Affiliations:** Centro TISP, Istituto Superiore di Sanità, 00161 Roma, Italy; daniele.giansanti@iss.it

*Assistive technologies, robotics, and automatic machines* are becoming important elements of the human health domain. The World Health Organization (WHO) is beginning to address the theme of *assistive technologies (ATs)* [1]. According to the WHO, “assistive technology” is an umbrella term covering systems and services related to the delivery of assistive products and services. The tools for AT can maintain or improve human independence and well-being, and these tools have very broad applications in the field of human health. These applications range from pill organization and spectacles up to communication aids, prostheses, wheelchairs, and many other tools. Such tools can also widely use IT and mechatronics and show different levels of automation. Nowadays, more than 1 billion people require at least one AT tool, but with the increase in world population, in 8 years, more than 2 billion people will require such tools. ATs allow people to become healthy, independent, productive, and to participate in all common activities in society (e.g., education, work, civil life). Therefore, the provision of these tools is essential. Unfortunately, today, AT tools are successfully accessed by only 1 in 10 people in need [1]. People who are most in need of ATs are [1,2]:

- People with gradual functional decline;
- Older people;
- People with communication problems;
- People with mental health disabilities;
- People with a wide range of disabilities.

The WHO identified five major *challenges* at the international level [1]:

(1) *The policy*. In most regions of the world, a policy on the ATs is lacking.
(2) *The products*. Unfortunately, the industry is particularly focused on high-income markets. However, these tools should be user-centered and tailored to one’s individual needs. According to *the International Classification of Functioning, Disability and Health* [3], two individuals with the same apparent disability are not identical.
(3) *The provision*. In high-income countries, services are not well-integrated but are nearly always independent. For example, multiple appointments at different locations are needed, which is time-consuming, stressful and tiring for both the citizen and the caregiver.
(4) *The personnel.* Trained personnel are crucial for important practices, such as the correct prescription of an AT tool, providing training, and organizing follow-up appointments for the user.
(5) *Assistive technology within universal health coverage.* The 2030 *Agenda of WHO for Sustainability* emphasizes universal health coverage so that everyone in the world can access both services and products without economic restrictions.

Today, the innovative prospects, usefulness and diffusion of *robotics in the health domain* are unconfutable.

The Policy Department for Economic, Scientific and Quality-of-Life Policies of the European Parliament identified the most important applications of care robots [4] to be the following:

- The robotic surgery;
- The care and socially assistive robotics;
- The robotic rehabilitation;
- The robotics for training in the health domain.

The debate on both the successes and failures of these tools is widespread both in their industrial use [5,6] and in healthcare [7]. The use of care robots seems to have important advantages for both all those involved in the *health domain* and their users [8].

For example, in surgery, robotics has reduced the probability of infection, blood deficit, and decreased recovery time. The application of care robots in rehabilitation and assistance programs optimizes care and minimizes workload. The general consensus is that care robots are effective, useful, and can work tirelessly. The current use of care robots includes the following [7,8]:

*Surgeries*—Care robots allow less invasive clinical interventions.

*Clinical training*—Robotics allow realistic simulations with force feedback due to their haptic technology.

*Prescription/dispensing*—Special robots can carry out the following with a high precision, accuracy and speed: (a) dispense drug treatment; (b) manage problematic liquids or viscous materials.

*Care/services*—Dedicated robotic systems can perform daily actions (for example, patient displacement) and daily measurement checks (e.g., pressure, temperature, glycemia).

*Disinfection and sanitation*—These robots carry out important routine activities in healthcare environments, such as disinfection processes and the air ventilation.

*Telepresence*—These care robots are properly configured with features related to telemedicine, eHealth and domotics, interacting with patients and/or providing ATs, integrating them into an ambient-assisted living.

*Logistic use*—Logistics robots perform basic tasks, such as transporting lunches or drug treatments.

*Rehabilitation and assistance*. The use of care robots to guide the patient in physiotherapy tasks is becoming the subject of increasing research interest. Additionally, the use of robots as companions or for psychological support seems to be very attractive [9]. Moreover, the use of robots as complex mechatronic–IT aids is very promising in the field of the above-described ATs.

Beside the innumerable benefits of these robots, there are also problems. For example, there is a probability of faults due to human error or mechatronic deficits. A single fault could cause physical or psychological damages/harms. Another important problem is the element of cost. Today, the use of care robots is mainly limited to first-world countries. Other problems manifest in the strong impact and implications of ethical concerns in this field [10,11].

These concerns mean that a strong and widespread acceptance of the robots’ integration in the health domain is necessary, with the need to obtain consensus initiatives, such as the *Consensus Conferences* [8], which is capable of producing strategic documents, as in the case of rehabilitation in Italy [12].

The introduction of *automatic machines* is radically changing the healthcare landscape with regard to decision-making, therapeutic, and rehabilitation approaches.

Important ethical issues are also being raised [13]. The impact of these machines based on artificial intelligence in the *health domain* is both significant and problematic at the same time, as they have the potential to halter the traditional healthcare–patient relationship, currently centered on the faith and openness of medical opinion and curative decisions. Through algorithms based on artificial intelligence, automatic machines can sometimes make decisions that are not fully transparent [13,14,15,16]. Therefore, there is a strong need for transparent approaches in data science, not only to the design of algorithms but also to the insiders, i.e., the clinicians. Automatic machines are increasing their applications in healthcare, for example, in the form of the following: (a) Detecting previously unidentified interferences that reduce the probability of adverse effects in drug interactions [14,15,16]; (b) integration into digital pathology and digital radiology [17,18,19]; and (c) working in direct contact with patients in rehabilitation and with social robots, with new practical and ethical implications [20].

We have briefly highlighted the peculiarities of the introduction of *assistive technologies, robotics, and automatic machines into the health domain*. In the healthcare process, these tools can represent single elements but can also be integrated in a cascade; the access to a robot or automatic machine, for example, can be supported in a disabled person by an AT. Assistive technologies, robotics, and automatic machines can also be integrated; think of an AT based on robotics with internal interactive processes derived from the automatic machine learning of artificial intelligence. There is a lot of research for scholars to carry out in the development of these tools in clinical applications. There is also a lot of study and work for ethicists, legislators, economists, and stakeholders.

It will be necessary to allow these solutions to be used for the benefit of an ever-wider audience of citizens; at the same time, it will be necessary to develop a targeted consensus and acceptance initiatives so that their introduction as single, integrated, or coincident elements takes place without trauma or shocks.

With this Special Issue, entitled “Assistive Technologies, Robotics, and Automated Machines in the Health Domain” (https://www.mdpi.com/journal/healthcare/special_issues/Assistive_Technologies_Robotics_Automated_Machines_Health_Domain [21] (accessed on 24 May 2022)), which has just opened, we intend to provide important contributions to this field by creating a forum that showcases the wide-ranging experience of its leading researchers.
